# Factors associated with preventive behaviors for COVID-19 infection among healthcare workers by a health behavior model

**DOI:** 10.1186/s41182-022-00454-z

**Published:** 2022-09-07

**Authors:** Chutatip Limkunakul, Sadiporn Phuthomdee, Palakorn Srinithiwat, Sasinun Chanthanaroj, Watchara Boonsawat, Kittisak Sawanyawisuth

**Affiliations:** 1 Department of Internal Medicine, Panyananthaphikkhu Chonprathan Medical Center, Srinakharinwirot University, Nonthaburi, Thailand; 2 Department of Academic and Research, Panyananthaphikkhu Chonprathan Medical Center, Srinakharinwirot University, Nonthaburi, Thailand; 3 Department of Medicine, Faculty of Medicine, Khon Kaen University, Khon Kaen, Thailand

**Keywords:** Knowledge, Perception, Prevention, COVID-19

## Abstract

**Background:**

Coronavirus disease 2019 (COVID-19) is a pandemic disease. There are limited data on predictors of good preventive behaviors among healthcare workers. This study aimed to evaluate if any factors were predictors of good preventive behaviors in healthcare workers under the theory of health behavior model.

**Methods:**

This was a cross-sectional study in healthcare workers who were willing to participate in the study. Participants were requested to fill out a self-administered questionnaire that comprised health behavior model and preventive behaviors from COVID-19 infection. Factors associated with preventive behavior, an outcome, were analyzed by multivariate linear regression analysis.

**Results:**

There were 273 healthcare workers who participated in this study. The average (SD) age and working duration of participants was 38.9 (12.1) and 11.4 (9.8) years. The preventive behavior category had an average score of 87.6% (70.3/80). After adjusted, knowledge and perception of personal preventability were independently associated with preventive behaviors. The adjusted coefficients of both factors were—0.911 (*p* 0.009) and 0.477 (*p* < 0.001).

**Conclusions:**

Specific knowledge and perception of personal ability questions were associated with preventive behaviors for COVID-19 infection. To improve personal preventive behaviors in healthcare workers, these factors should be emphasized.

**Supplementary Information:**

The online version contains supplementary material available at 10.1186/s41182-022-00454-z.

## Introduction

Coronavirus disease 2019 (COVID-19) is a pandemic disease with mortality rate of 45% if invasive mechanical ventilator is required [1]. One risk factor of COVID-19 infection is occupation [2]. Healthcare workers (HCWs) are one occupation recognized as high-risk group for COVID-19 infection [2]. The database study found that health care practitioners had the highest case count at 1,208 cases followed by transportation and material moving at 1096 cases, healthcare support at 989 cases, and production at 964 cases [2]. There were 114,529 HCWs in the US infected with COVID-19 resulting in 574 deaths [3]. Additionally, HCWs may experience psychiatric conditions. A study from Ecuador found that HCWs reported depression in 27.3% and anxiety in 39.2% during the COVID-19 pandemic [4]. Even though COVID-19 vaccination is important, preventive behaviors remain necessary.

Knowledge, attitude, and practice are key factors for COVID-19 prevention. A study from China and United Arab Emirates found that college students and HCWs had knowledge of COVID-19 infection of 73.81% and 61% [5, 6]. A study on general population found that women and other factors such as perceived benefits were associated with preventive behaviors [7]. Health belief model (HBM) by Rosenstock et al. is a widely accepted and popular theoretical guideline for health behaviors in public health research. It contains the perception of susceptibility, severity, benefits, barriers, health behaviors and cues to action [8, 9]. We used this model for construct our questions in the survey to explore reasons why HCWs have COVID-19 infection prevention behaviors. There are limited data on predictors of good preventive behaviors in healthcare workers. This study aimed to evaluate if any factors were predictors of good preventive behaviors among HCWs.

## Methods

This was a cross-sectional study conducted at Panyananthaphikkhu Chonprathan Medical Center, Nonthaburi, an affiliated Hospital to Srinakharinwirot University, Thailand. The inclusion criteria were HCWs who were willing to participate in the study. The HCWs included nurses, nurse assistants, medical service staff, medical technologists, dentists, dentist assistants, pharmacists, pharmacist assistants, physical/occupational therapists, radiological technologist, medical scientists, public health staff, patient assistants, and office staffs. The study period was between September and October 2020.

Participants were randomly selected from the hospital personnel list and requested to fill out a self-administered questionnaire. The questionnaire comprised nine sections: baseline characteristics, knowledge (10 items), perception towards risky possibility of COVID-19 infection (10 items), perception of COVID-19 severity (10 items), benefits of abiding by medical advice on COVID-19 preventive measures (12 items), barriers of prevention strategies of COVID-19 infection (12 items), factors leading to COVID-19 preventive behaviors (12 items), perception of personal preventability (12 items), and preventive behaviors from COVID-19 infection (16 items). The knowledge part was a yes/no question, while the preventive behaviors had five responses as always, often, sometimes, rarely, and never. The other categories had five Likert rating scales: strongly agree, partially agree, neutral, partially disagree, and strongly disagree. The knowledge part had a total score of 10, while the other part had scores according to the scale from 1 to 5. The questionnaire was checked for content validity and reliability. An item-objective congruence and the Cronbach’s alpha reliability coefficient of the questionnaire were 0.86 and 0.88, respectively.

*Sample size calculation.* A previous study found that 98% of participants had preventive behavior, while we expected that 95% of participants had preventive behavior [10]. For a single study group with an alpha error of 5% and power of 80%, the required sample size was 233 participants. The questionnaire was distributed randomly to approximately 30% of total HCWs of 1060 persons.

*Statistical analyses.* Descriptive statistics were used to calculate mean (SD) and number (percentage) of variables in the questionnaire. Scores of knowledge (10 points), perception of risk factors of COVID-19 infection (50 points), perception of severity of COVID-19 infection (50 points), benefits of following medical advices of COVID-19 infection (60 points), barriers of prevention strategies of COVID-19 infection (60 points), factors associated with preventive behaviors of COVID-19 infection (60 points), perception of personal ability to prevent COVID-19 infection (60 points), and preventive behaviors (80 points) were calculated for each participant. Factors associated with preventive behavior, an outcome, were executed by multivariate linear regression analysis. Questions in the significant parts were also executed for prediction of preventive behavior score by linear regression analysis. The analyses were performed by STATA software, version 10.1 (College Station, Texas, USA).

## Results

There were 273 HCWs that participated in this study. An average (SD) age and working duration of participants was 38.9 (12.1) and 11.4 (9.8) years. Most participants were female (88.8%), holding bachelor degree (58.6%), and had job title as nurse (36.6%) as shown in Table 1. Additional file 1, comprising seven tables, shows details of responses to seven categories of the questionnaire. The outcome, preventive behaviors in healthcare workers, is shown in Table 2. The participants had average knowledge score (SD) of 7.0 (1.3)/10, while the perception of personal preventability category had the highest average score (SD) of 49.2 (6.2)/60 (Table 3). The preventive behavior category had an average score of 87.6% (70.3/80), as shown in Table 3.

**Table 1:** Characteristics of healthcare workers who participated in the COVID-19 preventive behavior project (*n* = 273)

Factors	Number (%)
Age, years	38.9 (12.1)
Gender, female	238 (88.8)
*Education*	
Lower than bachelor’s degree	96 (35.8)
Bachelor’s degree	157 (58.6)
Master’s degree or PhD	15 (5.6)
Working experience, years	11.4 (9.8)
*Job title*	
Nurse	100 (36.6)
Office staff	75 (27.5)
Medical service staff	34 (12.5)
Nursing assistant	28 (10.3)
Medical technologist	7 (2.6)
Dental assistant	6 (2.2)
Pharmacist	5 (1.8)
Patient care assistant	4 (1.5)
Physical/occupational therapist	4 (1.5)
Dentist	3 (1.1)
Pharmacist assistant	3 (1.1)
Radiological technologists	2 (0.7)
Medical scientist	1 (0.4)
Public health staff	1 (0.4)

**Table 2:** Healthcare workers responses to questions regarding preventive behaviors in healthcare workers

Questions	Always	Often	Sometimes	Rarely	Never
1. You regularly wear a surgical mask every time you are close with patients or take care of high-risk contact	91.5, 248	7.0, 19	0.7, 2	0.7, 2	–
2. You regularly wear a surgical mask every time you are in the community space or outbreak areas	93.0, 252	6.3, 17	0.7, 2	–	–
3. You regularly wash hands with soap and water or alcohol gel before and after exposing a close contact with patients or staying in outbreak areas	83.8, 227	12.5, 34	2.2, 6	0.7, 1	0.7, 1
4. If you stay in the outbreak areas, you always wear surgical or cloth masks	87.8, 238	11.1, 30	0.7, 2	0.4, 1	–
5. Your workplace provides you with enough sanitizing devices and surgical masks	69.0, 187	19.2, 52	6.6, 18	3.7, 10	1.5, 4
6. Your workplace arranges social distancing in sharing space, such as elevators and canteen, and you abide well with the policy	70.5, 191	24.4, 66	4.1, 11	1.1, 3	–
7. You sanitize your workplace with antiseptics, detergent, and alcohol	63.8, 173	29.2, 79	5.2, 14	1.8, 5	–
8. You measure your body temperature before entering the community areas	87.5, 237	10, 27	2.2, 6	0.4, 1	–
9. You properly dispose of used surgical masks by tightly wrapping them before putting it in the plastic bag before putting it in the trash	37.9, 184	23.2, 63	6.3, 17	1.1, 3	1.5, 4
10. When you talk with COVID-19 patients or high-risk contact, you will leave at least 1–2 m distance	60.1, 163	28.8, 78	7.4, 20	1.5, 4	2.2, 6
11. You avoid having meals in the restaurants of infectious risk	53.9, 146	26.6, 72	12.5, 34	3.7, 10	3.3, 9
12. You take a shower immediately after getting back home	56.1, 152	30.6, 83	10.3, 28	2.2, 6	0.7, 2
13. You regularly take 30-min exercise at least three times a week, have healthy meals, use personal utensils and have enough sleep for 6–8 h per day to decrease COVID-19 infection risk	21.4, 58	31.7, 86	33.9, 92	11.1, 30	1.8, 5
14. You suggest COVID-19 patients wearing surgical mask when you are surrounded with high-risk contact or stay in outbreak areas	46.9, 127	31.4, 85	13.7, 37	1.8, 5	6.3, 17
15. You suggest COVID-19 patients creating social and family isolation	33.9, 92	31.7, 86	17.7, 48	4.8, 13	11.8, 32
16. As a healthcare worker, you take care of COVID-19 patients or high-risk contact using preventive devices and PPE	36.5, 99	18.5, 50	12.5, 34	5.2, 14	27.3, 74

Data presented as percentage, number

**Table 3:** Frequency distribution of mean, standard deviation and standardized mean of health belief model constructs for preventive behaviors of COVID-19 infection in healthcare workers

	No.	Range	Mean	SD	Min	Max
Knowledge	10	0–1	7.0	1.3	4	10
Perception towards risky possibility of COVID-19 infection	10	1–5	39.6	4.6	27	50
Perception of COVID-19 severity	10	1–5	39.4	4.1	26	50
Benefits of abiding by medical advice on COVID-19 preventive measures	12	1–5	45.6	8.3	16	60
Barriers of prevention strategies	12	1–5	43.9	5.0	30	60
Factors leading to COVID-19 preventive behaviors	12	1–5	48.5	6.3	32	60
Perception of personal preventability	12	1–5	49.2	6.2	34	60
Preventive behaviors	16	1–5	70.3	6.8	45	80

Among studied variables, there were five factors significantly associated with preventive behaviors by univariate linear regression analysis including perception towards risky possibility of COVID-19 infection (coefficient 0.335; *p* < 0.001), barriers of prevention strategies (coefficient 0.343; *p* < 0.001), benefits of abiding by medical advice on COVID-19 preventive measures (coefficient 0. 279; *p* < 0.001), factors leading to COVID-19 preventive behaviors (coefficient 0.287; *p* < 0.001), and perception of personal preventability (coefficient 0.481; *p* < 0.001), as shown in Table 4. After adjusted, knowledge and perception of personal preventability were independently associated with preventive behaviors. The adjusted coefficients of both factors were − 0.911 (*p* 0.009) and 0.477 (*p* < 0.001), as shown in Table 4. One question in the knowledge part was negatively associated with preventive behavior score (coefficient of − 1.978; *p* 0.012), as shown in Table 5, while four questions in the perception of personal ability to prevent COVID-19 infection were positively associated with preventive behavior score (Table 6).

**Table 4:** Factors associated with preventive behaviors of COVID-19 infection by linear regression analysis in healthcare workers

Questions	Univariate analysis			Multivariate analysis		
	Coefficient	95% CI	*p* value	Coefficient	95% CI	*p *value
Knowledge	− 0.434	− 1.057, 0.189	0.171	− 0.911	− 1.587, − 0.234	0.009
Perception of risk factors	0.335	0.162, 0.509	< 0.001	0.108	− 0.109, 0.325	0.330
Perception of COVID-19 severity	0.152	− 0.045, 0.349	0.129	− 0.021	− 0.253, 0.212	0.860
Barriers of prevention strategies	0.343	0.184, 0.503	< 0.001	0.073	− 0.141, 0.287	0.502
Benefits of abiding by medical advice on COVID-19 preventive measures	0.279	0.186, 0.372	< 0.001	0.081	− 0.063, 0.225	0.269
Factors leading to COVID-19 preventive behaviors	0.287	0.162, 0.413	< 0.001	− 0.087	− 0.290, 0.115	0.395
Perception of personal preventability	0.481	0.362, 0.601	< 0.001	0.477	0.269, 0.685	< 0.001
Sex	0.661	− 1.956, 3.278	0.619	− 1.005	− 3.511, 1.501	0.430
Working experience	0.040	− 0.046, 0.127	0.356	− 0.003	− 0.144, 0.137	0.963
Age	0.032	− 0.037, 0.101	0.365	0.032	− 0.081, 0.145	0.577

*CI* confidence interval

**Table 5:** Factors associated with preventive behaviors of COVID-19 infection by knowledge questions in healthcare workers

Questions	Univariate analysis			Multivariate analysis		
	Coefficient	95% CI	*p* value	Coefficient	95% CI	*p* value
1. COVID-19 coronavirus causes disease in humans and infected people are mostly asymptomatic	0.322	− 1.143, 1.787	0.666	0.863	− 0.678, 2.403	0.271
2. COVID-19 can be transmitted person-to-person through droplets produced from cough, sneeze, or infectious contact via conjunctiva and nasal mucosa	− 1.598	− 8.161, 4.965	0.632	− 0.405	− 9.884, 9.074	0.933
3. COVID-19 coronavirus can be easily eliminated by washing and rubbing hands with soap and water for 20 s	− 0.875	− 2.361, 0.611	0.248	− 0.494	− 2.057, 1.069	0.534
4. Children are at higher risk of COVID-19 infection. If they are infected, their symptoms are always severe	− 1.890	− 3.359, − 0.421	0.012	− 1.978	− 3.515, − 0.422	0.012
5. You are at risk for COVID-19 if you have a close contact with patients or infected people within one-meter distance for 5 min	− 0.791	− 2.730, 1.149	0.423	− 0.412	− 2.483, 1.659	0.696
6. Symptoms of COVID-19 patients are a fever, cough, sneeze, and muscle pain. For severe cases, patients show symptoms of difficulty breathing that may lead to death	− 0.226	− 4.413, 3.960	0.915	− 0.508	− 4.970, 3.955	0.823
7. In case that you accidentally contact asymptomatic COVID-19 patients with no fever, cough, or sneeze; you are at low risk to infect	0.199	− 1.454 to 1.852	0.813	0.046	− 1.657, 1.750	0.957
8. Male COVID-19 patients have more severe symptoms than female	− 0.571	− 2.147, 1.006	0.477	− 0.638	− 2.284, 1.007	0.446
9. Washing hands with any concentrated alcohol gel can kill COVID-19 coronavirus	− 1.285	− 2.950, 0.381	0.13	− 1.365	− 3.093, 0.362	0.121
10. Solely wearing a surgical mask definitely prevents COVID-19 infection	1.194	− 1.517, 3.905	0.387	1.843	− 1.088, 4.774	0.217

*CI* confidence interval

**Table 6:** Factors associated with preventive behaviors of COVID-19 infection by questions on perception of personal preventability to prevent COVID-19 infection in healthcare workers

Questions	Univariate analysis			Multivariate analysis		
	Coefficient	95% CI	*p* value	Coefficient	95% CI	*p* value
1. You can actually wear a surgical mask covering your mouth and nose in public	1.443	0.166, 2.721	0.027	− 0.671	− 2.030, 0.689	0.332
2. You have no ability to convince people with COVID-19 high-risk contact to cover their mouth and nose while coughing or sneezing	2.074	1.319, 2.828	< 0.001	0.702	− 0.304, 1.708	0.171
3. You cannot convince persons at risk of COVID-19 infection to seek for medical attention	1.890	1.226, 2.554	< 0.001	0.975	0.081, 1.868	0.033
4. You frequently wash hands with soap and water or rub alcohol gel after contacting with suspected infective surroundings outside the household	2.659	1.478, 3.840	< 0.001	0.695	− 0.662, 2.051	0.314
5. You have no ability to suggest COVID-19 patients creating social or family isolation	1.825	1.076, 2.575	< 0.001	0.233	− 0.734, 1.200	0.636
6. It is unnecessary to use protective devices, such as surgical masks, for patients with mild cases or high-risk contact	1.242	0.390, 2.094	0.004	− 0.840	− 1.939, 0.259	0.133
7. You cannot separately have meals from asymptomatic COVID-19 carriers	1.481	0.673, 2.289	< 0.001	0.203	− 0.814, 1.220	0.695
8. You can stay healthy by eating healthy meals, using your own utensils, exercising for thirty minutes three times a week and getting enough sleep	2.152	1.349, 2.956	< 0.001	0.720	− 0.184, 1.624	0.118
9. You cannot avoid staying in crowded places	1.885	1.216, 2.553	< 0.001	0.950	0.205, 1.624	0.013
10. After the exposure of high-risk contact, you cannot access in COVID-19 screening test	2.001	1.335, 2.666	< 0.001	0.937	0.149, 1.726	0.020
11. You can disinfect your residence by hygienization with detergent, water, and hypochlorite	2.615	1.664, 3.566	< 0.001	0.505	− 0.618, 1.629	0.377
12. You can properly dispose of surgical masks by wrapping them before putting it in the trash with a closed lid	2.243	1.371, 3.116	< 0.001	0.990	0.044, 1.937	0.040

*CI* confidence interval

## Discussion

Specific knowledge question and perception of personal preventability were found to be predictors of good preventive behaviors for COVID-19 infection among HCWs in this study.

A study from Bangladesh conducted in the general population found that higher knowledge scores were positively associated with higher preventive behaviors for COVID-19 (beta 0.053; *p* < 0.001) as well as in HCWs [7, 11–13]. Despite the average knowledge score and preventive behavior were not different from general population (70.00% vs 71.82%) and (87.88% vs 84.75%), respectively, we found the opposite results (Table 4) [14]. Our study showed that non-medical doctor (MD) HCWs with good knowledge score had lower preventive behavior score (beta 0.911; *p* 0.009). These findings may explain from difficulty of our questions. From our analysis, those with high preventive behavior had low score on question no. 4 in the questionnaire with a coefficient of − 1.978 (*p* 0.012). Since COVID-19 infection is an emerging disease, we have gap of knowledge among infected children at that period of time. Also, there were few cases of COVID-19-infected children reported in Thailand during the study period, Subsequently, the low score on the question about children resulting in negative association with the preventive behavior. Other possible explanations are the shortage of personal protective equipments at that period of time for whole country and finally HCWs may feel difficult to change their habits as previously reported [12].

There were four questions regarding perception of personal ability to prevent COVID-19 infection, including seeking medical attention, crowded places, surveillance test after exposure, and disposal of protective equipment (Table 6; Additional file 1). These perceptions have been reported to be associated with preventive behaviors for COVID-19 infection in HCWs [15, 16]. From our analysis, factors on perception are more important than knowledge on practice of prevention. In other words, personal attitude may be crucial for prevention practice in COVID-19 infection. These findings may be different from a study from Vietnam which reported that knowledge was a significant factor associated with practice level (p 0.00) but not barriers of practice (*p* 0.056) [12]. It might be due to differences on questions in the questionnaire. Further studies are required to conclude this issue.

Four questions about perception of personal ability to prevent COVID-19 infection which significantly associated with preventive behaviors should be concerned (Table 6). Our suggestions were HCWs should encourage persons at risk of COVID-19 to seek medical attention. The hospital should sufficiently provide surveillance test opportunities after exposure with persons at risk as well as endow with safe management system for infectious waste disposal. Finally, HCWs essentially use personal protective personal protective equipments (PPEs) in public area. This may contribute to improving good practices and preventive behavior among HCWs in particular. However, even with PPEs, some HCWs may still be at risk for COVID-19 infection such as dentists or nurses. These two occupations had prevalence ratio of 2.62 and 2.61 with 95% confidence interval of 1.23–4.02 and 1.16–4.14, respectively [2].

Our study had some limitations. First, the study population was HCWs in affiliated hospital of medical school, who are non-MD staffs. The findings of this study may not be generalizable for all HCWs in other hospital levels. However, this questionnaire was already validated. Second, the data were collected after the third wave of epidemic period in Thailand. Consequently, the results may vary with the stage of COVID-19 outbreak and the method of data collection is based only on the responses to the developed questionnaire which cannot verify the truth of the answer. Note that no specific clinical risk factors are evaluated such as obstructive sleep apnea [17–22]. Finally, there might be might be cultural differences or norm with other population such as general population [11, 13].

In conclusion, one knowledge question and four questions related to the perception of personal ability were significantly associated with preventive behaviors for COVID-19 infection. To improve personal preventive behaviors in healthcare workers, these factors should be emphasized.

## Supplementary Information

**Additional file 1.** Responses to the quesionnaire of health belief model on COVID-19 infection in healthcare workers.

## Data Availability

The data relating to this manuscript are available upon request.
