# Supplementary material for: Factors associated with preventive behaviors for COVID-19 infection among healthcare workers by a health behavior model
Source: Trop Med Health. 2022 Sep 7;50:65. doi: 10.1186/s41182-022-00454-z (PMC9449286; doi:10.1186/s41182-022-00454-z)
Supplement: Supplementary file 1 — Additional file 1. Responses to the quesionnaire of health belief model on COVID-19 infection in healthcare workers. [file 41182_2022_454_MOESM1_ESM.docx]

**Table 1** Healthcare Worker Responses to Questions Regarding Knowledge of COVID-19 Infection.

| **Questions** | **Yes** | **No** |
| --- | --- | --- |
| 1. COVID-19 coronavirus causes disease in humans and infected people are mostly asymptomatic. | 50.7, 138 | 49.3, 134 |
| 2. COVID-19 can be transmitted person-to-person through droplets produced from cough, sneeze, or infectious contact via conjunctiva and nasal mucosa. | 98.9, 268 | 1.1, 3 |
| 3. COVID-19 coronavirus can be easily eliminated by washing and rubbing hands with soap and water for 20 seconds. | 57.4, 156 | 42.6, 116 |
| 4. Children appear to be at higher risk for COVID-19 and more severe symptom. | 45.4, 123 | 54.6, 148 |
| 5. You are at risk for COVID-19if you have a close contact with patients or infected people within one-meter distance for 5 minutes. | 80.4, 217 | 19.6, 53 |
| 6. Symptoms of COVID-19 patients are a fever, cough, sneeze, and muscle pain. For severe cases, patients show symptoms of difficulty breathing that may lead to death. | 97.1, 264 | 2.9, 8 |
| 7. In case that you accidentally contact asymptomatic COVID-19 patients with no fever, cough, or sneeze; you are at low risk to infect. | 26.3, 71 | 73.7, 199 |
| 8. Male COVID-19 patients have more severe symptoms than female. | 27.7, 75 | 72.3, 196 |
| 9. Washing hands with any concentrated alcohol gel can kill COVID-19 coronavirus. | 29.3, 79 | 70.7, 191 |
| 10. Solely wearing a surgical mask definitely prevents COVID-19 infection. | 8.1, 22 | 91.9, 250 |

Note. Data presented as percentage, number.

**Table 2** Healthcare Worker Responses to Questions Regarding Perception towards Risky Possibility of COVID-19 Infection.

| **Questions** | **Strongly agree** | **Partially agree** | **Neutral** | **Partially disagree** | **Strongly disagree** |
| --- | --- | --- | --- | --- | --- |
| 1. Elderly people are at the highest risk of infection. | 52.4, 143 | 35.9, 98 | 7, 19 | 4.4, 12 | 0.4, 1 |
| 2. Females have higher risk than male. | 2.9, 8 | 6.6, 18 | 51.1, 139 | 30.1, 82 | 9.2, 25 |
| 3. You are at risk if you stay with asymptomatic persons in a closed or unventilated room. | 53.1, 145 | 37.7, 103 | 5.1, 14 | 3.7, 10 | 0.4, 1 |
| 4. You have lower risk of infection, if you have comorbid diseases such as diabetes, heart disease, or lung diseases although you have been in close contact with infected people. | 8.4, 23 | 19.4, 53 | 18.7, 51 | 36.6, 100 | 16.8, 46 |
| 5.You are at risk in living without hand washing, wearing surgical masks , or staying in outbreak surroundings. | 80.1, 218 | 16.2, 44 | 1.8, 5 | 0.4, 1 | 1.5, 4 |
| 6. You have no chance of causing an infection even if you participate in the same group eating without anyone coughing or sneezing. | 5.9, 16 | 6.6, 18 | 8.8, 24 | 37.7, 103 | 41.0, 112 |
| 7. There is no chance of COVID-19 transmission even if you sleep in the same room with asymptomatic carriers. | 3.3, 9 | 5.9, 16 | 12.5, 34 | 41.4, 113 | 37, 101 |
| 8. You have no chance to get infected if you touch only the outer surface of the surgical mask you are wearing. | 2.2, 6 | 7.7, 21 | 21.6, 59 | 21.6, 122 | 23.8, 65 |
| 9.You have an infection risk if you walk into the market or community mall without customer limitation. | 31.5, 86 | 42.9, 1170 | 13.6, 37 | 9.2., 25 | 2.9, 8 |
| 10. You have no chance to get infected even if you have a close contact with patients within two-foot distance for 2 minutes. | 2.2, 6 | 10.3, 28 | 28.9, 79 | 35.9, 98 | 22.7, 62 |

Note. Data presented as percentage, number.

**Table 3** Healthcare Workers Responses to Questions Regarding Perception of COVID-19 Severity.

| **Questions** | **Strongly agree** | **Partially agree** | **Neutral** | **Partially disagree** | **Strongly disagree** |
| --- | --- | --- | --- | --- | --- |
| 1. COVID-19 coronavirus causes respiratory tract disease which is less severe than seasons. | 5.9, 16 | 4.4, 12 | 10.3, 28 | 42.6, 116 | 36.8, 100 |
| 2. If you are infected, you will always show severe symptoms. | 2.6, 7 | 4.8, 13 | 29.8, 81 | 48.2, 131 | 14.7, 40 |
| 3. Only one COVID-19 patient at home can spread COVID-19 to other people leading to infection and illness in the household. | 25.4, 69 | 46.3, 126 | 14.3, 39 | 11.8, 32 | 2.2, 6 |
| 4. COVID-19 infection causes illness and decreases pulmonary function and causes respiratory failure. | 41.9, 114 | 50.4, 137 | 4.4, 12 | 2.6, 7 | 0.7, 2 |
| 5. If you are infected COVID-19, you will get public shaming. | 14.7, 40 | 31.3, 85 | 27.6, 76 | 18.8, 51 | 7.4, 20 |
| 6. COVID-19 infection and illness require you to reach for medical care as soon as possible. | 65.1, 177 | 30.1, 82 | 2.6, 7 | 1.1, 3 | 1.1, 3 |
| 7. Asymptomatic COVID-19 patients do not need to stop working. | 1.5, 4 | 3.7, 10 | 6.3, 17 | 27.9, 76 | 60.7, 165 |
| 8. Infected with COVID-19 costs a lot of money | 11.0, 30 | 23.2, 63 | 32.4, 88 | 26.1, 71 | 7.4, 20 |
| 9. Asymptomatic COVID-19 patients can continue with daily lives as normal. | 1.1, 3 | 12.9, 35 | 15.8, 43 | 40.4, 110 | 29.8, 81 |
| 10. Asymptomatic COVID-19 patients can mingle with or be close with your children or beloved ones. | 1.5, 4 | 2.6, 7 | 7, 19 | 25.7, 70 | 63.2, 172 |

Note. Data presented as percentage, number.

**Table 4** Healthcare Worker Responses to Questions Centering on Benefit of Abiding by Medical Advice on COVID-19 Preventive Measures.

| **Questions** | **Strongly agree** | **Partially agree** | **Neutral** | **Partially disagree** | **Strongly disagree** |
| --- | --- | --- | --- | --- | --- |
| 1. Wearing a surgical mask at all times to cover your mouth and nose can prevent COVID-19 infection from contact of phlegm, nasal discharge, and saliva from infected patients. | 54, 19.9 | 52.9, 144 | 15.4, 42 | 9.9, 27 | 1.8, 5 |
| 2. Recommending COVID-19 patients to cover mouth and nose or wear surgical mask while coughing or sneezing has no impact towards your prevention from being infected. | 4, 11 | 16.5, 45 | 15.4, 42 | 43.8, 119 | 20.2, 55 |
| 3. COVID-19 is preventable if patients receive proper suggestions for urgent medical care. | 39.9, 108 | 52.0, 141 | 4.8, 13 | 2.6, 7 | 0.7, 2 |
| 4. Wearing a face shield without a surgical mask definitely secures enough to prevent COVID-19 infection. | 2.2, 6 | 4.0, 11 | 17.3, 47 | 38.6, 105 | 37.9, 103 |
| 5. Recommending COVID-19 patients to create social and family isolation can prevent yourself from COVID-19 infection. | 22.8, 62 | 49.3, 134 | 15.8, 43 | 11.0, 30 | 1.1, 3 |
| 6. Having conversation within a 1-2 feet distance without wearing a surgical mask in a short period of time, for instance, 3-4 minutes, can lower the chance of COVID-19 infection. | 1.1, 3 | 8.8, 24 | 22.8, 62 | 48.5, 132 | 18.8, 51 |
| 7. Separating meals with asymptomatic COVID-19 patients reduces chances of infectious contact with phlegm, nasal discharge, and saliva from infected patients when they cough or sneeze. | 25.7, 70 | 47.1, 128 | 13.2, 36 | 12.5, 34 | 1.5, 4 |
| 8. Taking good care of your health on a regular basis: eating healthy meals, routine exercise, and healthy sleep, can prevent COVID-19 infection. | 18.8, 51 | 36.8, 100 | 26.5, 72 | 16.9, 46 | 1.1, 3 |
| 9. Avoid staying in crowded places or communities can prevent you from COVID-19 infection. | 15.5, 42 | 45.4, 123 | 25.1, 68 | 12.5, 34 | 1.5, 4 |
| 10.Screening test for COVID-19 after the exposure of high risk contact, do not minimize infectious risk of COVID-19 transmission to people you have close contact with. | 8.1, 22 | 33.6, 91 | 19.6, 53 | 31, 84 | 7.7, 21 |
| 11. Household hygienization with detergent, water, or hypochlorite is not effective enough to prevent unintentional COVID-19 transmission from outside into the house. | 6.3, 17 | 28, 76 | 36.5, 99 | 23.6, 64 | 5.5, 15 |
| 12. Wearing gloves at all times, if there is a risky contact with any surfaces in public places, can prevent COVID-19 infection better than frequent hand wash. | 2.2, 6 | 10.4, 28 | 20.7, 56 | 46.7, 126 | 20, 54 |

Note. Data presented as percentage, number.

**Table 5** Healthcare Workers Responses to Questions Regarding Barriers against Preventive Strategies during COVID-19 Pandemic.

| **Questions** | **Strongly agree** | **Partially agree** | **Neutral** | **Partially disagree** | **Strongly disagree** |
| --- | --- | --- | --- | --- | --- |
| 1. Wearing a surgical mask to cover the mouth and nose is uncomfortable because of unbearable difficulty in breathing. | 1.9, 5 | 10.4, 28 | 6.7, 18 | 53.7, 145 | 27.4, 74 |
| 2. It is difficult to suggest COVID-19 patients covering their mouth and nose or wearing surgical masks when coughing or sneezing. | 2.2, 6 | 14.8, 40 | 14.1, 38 | 51.1, 138 | 17.8, 48 |
| 3.When you find out the suspected COVID-19 patients, it is difficult to advise them to reach an urgent medical care. | 4.8, 13 | 11.9, 32 | 22.2, 60 | 46.7, 126 | 14.4, 39 |
| 4. Frequent hand washing with soap and water or alcohol gel after contact with the surrounding outside the household is time-consuming, burdensome, and expensive. | 2.2, 6 | 4.5, 12 | 4.5, 12 | 49.8, 134 | 39, 105 |
| 5.Recommending COVID-19 patients to create social and family isolation is difficult and wasteful. | 2.6, 7 | 10.8, 29 | 23.4, 63 | 48, 129 | 15.2, 41 |
| 6. Separated bed in mild cases of COVID-19 patients is unpractical. | 1.8, 5 | 12.3, 33 | 14.1, 38 | 51.3, 138 | 20.4, 55 |
| 7. Separating meals with asymptomatic COVID-19 patients is difficult. | 2.2, 6 | 15.2, 41 | 13, 35 | 48.7, 131 | 20.8, 56 |
| 8. Taking good care of your health on a regular basis: eating healthy meals, routine exercise, and healthy sleep is time-consuming and wasteful. | 2.6, 7 | 4.5, 12 | 7.8, 21 | 52.8, 142 | 32.3, 87 |
| 9. Avoiding a crowded place or community is impossible for you. | 5.6, 15 | 17.1, 46 | 14.9, 40 | 41.3, 111 | 5.6, 15 |
| 10. Screening test for COVID-19 after the exposure of high risk contact is impractical because COVID-19 screening place is unknown and time-consuming and wasteful. | 3.7, 10 | 17.8, 48 | 16.7, 45 | 43.5, 117 | 18.2, 49 |
| 11. Household hygienization with detergent, water, or hypochlorite is wasteful and time-consuming. | 1.1, 3 | 7.4, 20 | 8.2, 22 | 53.9, 145 | 29.4, 79 |
| 12.Proper disposal of used surgical masks by wrapping them before putting it in the trash with a closed lid is unpractical. | 3.7, 10 | 8.9, 24 | 5.2, 14 | 49.8, 134 | 32.3, 87 |

Note. Data presented as percentage, number.

**Table 6** Healthcare Worker Responses to Questions of Factors Leading to COVID-19 Preventive Behaviors.

| **Questions** | **Strongly agree** | **Partially agree** | **Neutral** | **Partially disagree** | **Strongly disagree** |
| --- | --- | --- | --- | --- | --- |
| 1. Having COVID-19 patients in the same workplace does not affect your COVID-19 preventive behaviors. | 8.4, 23 | 23.1, 63 | 12.5, 34 | 37, 101 | 19, 52 |
| 2.Advices from medical staff/friends/family/media regarding COVID-19 patients care centering on transmission preventive approaches by encouraging patients to cover their mouth and nose while coughing and sneezing, as well as social isolation, have a great impact on your COVID-19 healthcare towards members in your household. | 37.4, 102 | 53.7, 146 | 7.4, 20 | 0.4, 1 | 1.1, 3 |
| 3. Surgical mask supply from government officers, friends, or family for preventive COVID-19 purpose has a great impact towards your actual preventive behavior. | 42.1, 115 | 53.5, 146 | 2.6, 7 | 1.1, 3 | 0.7, 2 |
| 4. Advices regarding frequent use of soap hand wash or alcohol gel from medical staff/friends/family/media as a COVID-19 infectious prevention have no impact towards your disinfection practice. | 4.8, 13 | 4.8, 13 | 7.3, 20 | 47.6, 130 | 35.5, 97 |
| 5. Alcohol/alcohol gel supply from government officers, friends, or family for frequent hand rub for preventive COVID-19 purpose has no impact towards your actual preventive behavior. | 1.8, 5 | 7.7, 21 | 8.1, 22 | 48.5, 132 | 33.8, 92 |
| 6. Accurate advices of household hygienizing with detergent or hypochlorite from government officers, friends, family, or media have a great impact towards your actual preventive behavior. | 33.3, 91 | 56.8, 155 | 6.2, 17 | 2.6, 7 | 1.1, 1 |
| 7. Advice regarding healthy behaviors on a regular basis: eating healthy meals, medium spoon appliance, routine exercise, and healthy sleep from government officers, friends, family, or media, can reduce COVID-19 infection chances leading to your actual preventive behavior. | 113, 41.4 | 51., 141 | 4.8, 13 | 1.5, 4 | 0.7, 2 |
| 8.Advices of screening test for COVID-19 after the exposure of high risk contact from government officers, friends, family, or media have no impact towards your actual preventive behavior. | 3.3, 9 | 10.7, 29 | 10.7, 29 | 49.6, 135 | 25.7, 70 |
| 9. The awareness of COVID-19 outbreak areas has no impact towards your actual preventive behavior or entry avoidance. | 5.9, 16 | 10.7, 29 | 6.3, 17 | 48.2, 131 | 29, 79 |
| 10.Quantity updates of infection, COVID-19 patients, and recovery have no impact towards your actual preventive behavior. | 4, 11 | 11.4, 31 | 9.6, 26 | 46.3, 126 | 28.7, 78 |
| 11. Treatment awareness or COVID-19 vaccination encourage you to abide by COVID-19 preventive measures. | 34.6, 94 | 54.4, 148 | 5.9, 16 | 4, 11 | 1.1, 3 |
| 12.Work-from-home policy or COVID-19 high risk contact emergency leave have a great impact towards your actual preventive behavior. | 38.2, 104 | 50.4, 137 | 9.2, 25 | 1.8, 5 | 0.4, 1 |

Note. Data presented as percentage, number.

**Table 7** Healthcare Workers Responses to Questions Regarding COVID-19 Personal Preventability.

| **Questions** | **Strongly agree** | **Partially agree** | **Neutral** | **Partially disagree** | **Strongly disagree** |
| --- | --- | --- | --- | --- | --- |
| 1. You can actually wear a surgical mask covering your mouth and nose in public. | 72.1, 196 | 26.5, 72 | 0.4, 1 | 0.4, 1 | 0.7, 2 |
| 2.You have no ability to convince people with COVID-19 high risk contact to cover their mouth and nose while coughing or sneezing. | 3, 8 | 5.5, 15 | 25.1, 68 | 45.4, 123 | 21, 57 |
| 3. You have no ability to convince people with COVID-19 high risk contact to get COVID-19 screening test. | 6.3, 17 | 8.5, 23 | 21.1, 57 | 45.9, 124 | 18.1, 49 |
| 4. You frequently wash hands with soap and water or rub alcohol gel after contacting with suspected infective surroundings outside the household. | 61.8, 168 | 35.3, 96 | 1.5, 4 | 1.5, 4 | 0 |
| 5. You have no ability to suggest COVID-19 patients creating social or family isolation. | 4.1, 11 | 6.6, 18 | 31, 84 | 43.5, 118 | 14.8, 40 |
| 6. It is unnecessary to use protective devices, such as surgical masks, for patients with mild cases or high risk contact. | 2.2, 6 | 3.3, 9 | 4.4, 12 | 32.5, 88 | 57.6, 156 |
| 7. You cannot separately have meals from asymptomatic COVID-19 carriers. | 1.5, 4 | 4.8, 13 | 9.2, 25 | 40.1, 109 | 44.5, 121 |
| 8. You can stay healthy by eating healthy meals, using your own utensils, exercising for thirty minutes three times a week and getting enough sleep. | 35.3, 96 | 44.1, 120 | 16.5, 45 | 3.7, 10 | 0.4, 1 |
| 9. You cannot avoid staying in crowded places. | 37.1, 101 | 37.5, 102 | 14, 38 | 9.9, 27 | 1.5, 4 |
| 10. After the exposure of high risk contact, you cannot access in COVID-19 screening test. | 3.7, 10 | 12.5, 34 | 17.3, 47 | 43.4, 118 | 23.2, 63 |
| 11. You can disinfect your residence by hygienization with detergent, water, and hypochlorite. | 40.4, 110 | 50.7, 138 | 6.3, 17 | 2.6, 7 | - |
| 12. You can properly dispose of surgical masks by wrapping them before putting it in the trash with closed lid. | 44.3, 121 | 46, 125 | 6.6, 18 | 2.2, 6 | 0.7, 2 |

Note. Data presented as percentage, number.
